# Optimizing China’s carbon quota allocation for peak emissions: A novel AMLC framework tailored to regional dynamics

**DOI:** 10.1371/journal.pone.0321644

**Published:** 2025-04-23

**Authors:** Lanlan Lian, Xiaojun Ma, Yimeng Wang, Qingli Dong

**Affiliations:** 1 School of Information and Business Management, Dalian Neusoft University of Information, Dalian, China; 2 School of Statistics, Dongbei University of Finance and Economics, Dalian, China; 3 Science and Technology Department, Shijiazhuang Posts and Telecommunications Technical College, Shijiazhuang, China; 4 School of Economics and Management, Dalian University of Technology, Dalian, China; Shanghai University of Electric Power, CHINA

## Abstract

As China advances its carbon reduction efforts, the equitable and efficient allocation of initial carbon quotas among provinces has become increasingly critical. This study proposes a novel two-stage Adjusting Measures to Local Conditions (AMLC) framework that integrates regional development disparities and marginal abatement costs into the quota allocation process. Employing a PSO-LSSVM model for carbon emission estimation and a hierarchical clustering algorithm, this study categorizes 30 Chinese provinces into six distinct types, facilitating a regionally adaptive allocation strategy. Key findings include: (1) Between 2003 and 2022, carbon emissions exhibited a structural shift from energy-abundant, carbon-exporting provinces with inefficient industrial structures to energy-deficient, carbon-importing provinces, intensifying regional disparities. (2) Equity-based allocation schemes tend to favor regions with abundant carbon sinks, yet all provinces in the sixth category experience quota deficits; conversely, efficiency-based allocation disproportionately benefits initially efficient regions, exacerbating interprovincial disparities. (3) The AMLC framework enables 17 provinces across all clusters to achieve quota surpluses while significantly enhancing national green productivity, demonstrating its superiority in promoting high-quality economic development. These findings provide valuable insights for policymakers in designing macroeconomic strategies that balance equity and efficiency in carbon quota distribution, thereby supporting China’s transition toward peak emissions and sustainable growth.

## 1. Introduction

### 1.1. Background

Climate change is a global environmental crisis that profoundly affects ecological balance, human well-being, and socio-economic progress. The continuous increase in greenhouse gas emissions, especially carbon dioxide, is the primary catalyst for global warming [1], necessitating urgent and innovative mitigation strategies [2]. In this context, China, as the world’s largest emitter and a key participant in global climate policy, has committed to peaking its carbon emissions by 2030 [3], marking a critical step toward green, low-carbon development and sustainable modernization [4]. The carbon emissions trading scheme is considered a powerful regulatory mechanism to curb greenhouse gas emissions [5]. This mechanism treats carbon dioxide emission rights as special commodities, viewing future cumulative carbon emissions as a finite global resource that can be shared among regions [6]. Carbon quotas, as carriers and certificates of emission rights, represent the amount of carbon dioxide that regulated entities are allowed to emit [7].

**Fig 1:**
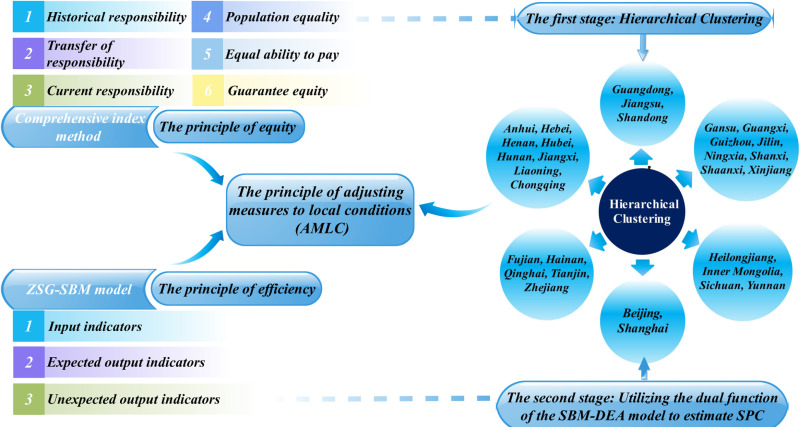
Diagram of the core structure of this study.

Scientifically allocating initial carbon quotas across regions is the primary and most crucial step in establishing a robust carbon emissions trading scheme [8]. A reasonable allocation not only facilitates the orderly construction of the carbon emissions trading market but is also closely linked to the achievement of overall carbon reduction goals. With the parallel operation of China’s eight pilot carbon markets and the national carbon market, the importance of inter-provincial initial carbon quota allocation has become increasingly prominent. China’s unique socialist market economy system and strong government guidance framework present inter-provincial allocation challenges different from those in the EU carbon market. Allocation schemes based on different criteria will affect the effective distribution of emission reduction costs among regulatory entities [9]. China’s vast territory and significant regional development disparities make it challenging to balance national strategic objectives with regional characteristics in the initial allocation of quotas. This study addresses this issue by proposing a quota allocation scheme tailored to local conditions, as outlined in the structure shown in Fig 1.

### 1.2. Literature review and research gaps

With the announcement of China’s “dual carbon” goals, the issue of inter-provincial initial carbon emission rights allocation has garnered widespread attention from scholars. The core of the debate centers around issues of equity and efficiency.

*Equity in Carbon Allocation* The principle of equity, foundational to the global climate response, necessitates a fair distribution of carbon emission space. Teng et al. highlight equity as a cornerstone principle, essential for a just allocation framework. Furthering this discourse [10], Clarke-Sather et al. employed statistical measures such as the coefficient of variation, Thiel index, and Kakwani index to assess the equity of carbon emission distribution among China’s provincial regions, and the results show that the level of carbon equity is lower in the central and western regions [11]. De Villemeur and Leroux employed the concept of equity to assign the cost of global warming as a collective responsibility across all economies [12]. At the same time, the indicator method is often applied to quota scheme design under the principle of equity, including the single-indicator method [13] and the composite indicator method [14]. To quantify the level of regional carbon equity from different perspectives, existing studies often refer to grandfather theory, polluter pays theory, egalitarian theory, and economic activity theory to select indicators [15]. However, the use of the indicator method will inevitably involve the issue of “strong subjectivity”. Existing studies often select a limited number of frequently used indicators, such as historical carbon emission responsibility and population size, in a fragmented manner to construct evaluation frameworks. This approach tends to be one-sided and lacks representativeness. Therefore, selecting a comprehensive and well-rounded set of equity principle indicators will be a key point for future research.

*Efficiency in Carbon Allocation* Parallel to equity, the efficiency principle advocates for allocation based on economic efficiency in reducing carbon emissions, positing that regions with higher carbon efficiency should yield higher economic outputs for the same level of emissions [16,17]. Existing studies often use the proportional relationship between inputs and outputs as a measure of economic efficiency, and data envelopment analysis (DEA) is favored due to its ability to reflect the efficiency of resource allocation from both the input and output perspectives. Given the fixed total carbon quota, interprovincial allocation games are inevitable, and scholars often rely on the zero-sum gains DEA (ZSG-DEA) model to allocate carbon quotas under the principle of efficiency. Chen et al. constructed the ZSG-DEA model for quota allocation based on the super-SBM model to measure efficiency, ensuring that the allocation results obtained by each provincial region are more accurate and effective [18]. Yang et al. enhanced the iterative method of the traditional ZSG-DEA model for optimization purposes and gave the optimal adjustment values for each scenario to guide the interprovincial quota allocation in China in 2030 [19]. However, based on the expression of the ZSG-DEA model, it does not sufficiently account for the impact of external environments on the efficiency of decision-making units. Its consideration of issues such as factor slack is also limited, ultimately leading to significant deviations between the allocation results and real-world data.

*Towards a Comprehensive Allocation Model* The binary focus on either equity or efficiency has yielded allocation outcomes that sometimes undermine collective economic interests or impede regional emission reduction initiatives [20]. Recognizing this, a new wave of scholarship advocates for a balanced model that integrates both principles. Wang and Kong employed the entropy weight method to integrate carbon quota allocation results based on the principles of equity and efficiency, resulting in a comprehensive allocation scheme. Their study found that the improved scheme, due to smaller differences in abatement costs across regions, was more readily accepted by a broader range of stakeholders [21]. Chen et al. integrated the equity principle’s basic indicators with the overall efficiency indicators derived from the Super-SBM model, and they used them as a reference standard for interprovincial quota allocation [22].Tian et al. set up four preference scenarios based on the equity indicator system and built a CCR-DEA model to evaluate the efficiency of each scenario under the assumption of constant returns to scale [23]. These models have demonstrated potential in surpassing national planning schemes in both equity and effectiveness [24].

In summary, allocation schemes based on the comprehensive principle often integrate indicators under the principles of equity and efficiency as the basis for quota distribution. However, since equity emphasizes the equal distribution of resources, while efficiency focuses on optimal allocation, combining these inherently conflicting factors makes it difficult to find a true balance. Furthermore, such schemes typically apply uniform allocation standards across regions, disregarding regional development differences and showing low sensitivity to changes in local carbon policies and economic conditions. These shortcomings highlight the need for differentiated quota strategies tailored to regional characteristics to avoid the inequities caused by one-size-fits-all standards and to enhance policy adaptability.

### 1.3. Innovation point

Based on the existing research gaps, this paper makes the following innovative contributions. (1) It expands the connotation of the equity principle and applies a more realistic ZSG-SBM allocation model under the efficiency principle, accounting for factor slack in undesired outputs. (2) A two-stage AMLC (Adjusting Measures to Local Conditions) principle is proposed, integrating regional disparities and marginal abatement costs into quota allocation. In the first stage, 30 provinces and municipalities are grouped using a clustering algorithm, with quotas allocated based on equity indicators. In the second stage, shadow price theory is used to measure the marginal abatement costs of regions, prioritizing quota support for those with higher costs. The necessity of the AMLC principle is confirmed by comparing quota allocation results under the carbon peak scenario. However, the study mainly focused on land-based carbon sinks, overlooking ocean carbon sinks, which could lead to incomplete assessments, especially for coastal regions. Additionally, reliance on existing data may limit the AMLC principle’s adaptability in dynamic contexts.

The paper is structured as follows: Section 2 outlines the improved initial quota allocation scheme, Section 3 analyzes spatial variation in regional development in China, Section 4 implements quota allocation under the “carbon peaking” scenario and selects the optimal scheme, and Section 5 presents conclusions and policy recommendations.

## 2. Improved initial quota allocation scheme

### 2.1. Refined equity principle

The Equity Principle, widely recognized in quota allocation as a key framework for distributive justice [25], is often inadequately addressed in existing research, with definitions and measures of carbon equity frequently shaped by subjective preferences. This study responds by integrating and extending prior work, offering a more comprehensive interpretation of the Equity Principle across six dimensions.

#### 2.1.1. Influencing factors and indicator selection for equitable distribution.

A rational delineation of regional emission reduction responsibilities is crucial for equitable quota allocation. Scholars often assess these responsibilities using historical responsibility, typically quantified by cumulative carbon emissions, arguing that regions with greater economic benefits should bear more responsibility and receive fewer quotas [26,27], in line with the polluter pays principle. In global value chains, regional trade conceals significant carbon transfers, necessitating their exclusion when assessing emission reduction responsibilities. Zhou et al. indirectly measured transferred carbon emissions to reflect actual levels in each province [28]. However, few studies address current carbon responsibilities, which are influenced by industrial and energy structures. This study uses the Index of Industrial Structure Rationalization and Proportion of Coal Consumption to assess these factors, both inversely related to quota allocation—lower values indicate more efficient structures, less energy use, and higher quota allocations.

In addition to responsibility factors, population equality, payment capacity, and guarantee fairness influence equitable distribution. Kverndokk argued that population-based quota allocation is both fair and feasible [29], with population size positively correlated with quota amounts [30]. Equality of payment capacity considers economic entities’ ability to bear costs, as reflected by per capita disposable income, which is also positively correlated with quotas [31]. Tian et al. identified forest stock volume and sown crop area as indicators of guarantee fairness, given their carbon sequestration attributes, further positively influencing quota allocations [23].

Based on these factors, an indicator system is constructed, as shown in Table 1, focusing on 30 provincial-level regions in mainland China from 2003 to 2022 (excluding Hong Kong, Macau, Taiwan, and Tibet due to data limitations). Nominal price data have been deflated to 2003 real values. The selection of the study period is primarily based on data availability and reliability. 2003 marks the beginning of systematic recording and disclosure of carbon emission-related data in China. Before this, carbon emission data were fragmented, lacking continuity and comparability. From 2003 onward, institutions such as the National Bureau of Statistics and the National Energy Administration progressively improved their data collection and reporting mechanisms, ensuring greater consistency and accuracy. Selecting 2003 as the starting point allows us to leverage a comprehensive and scientifically rigorous dataset, enhancing the reliability of our analysis.

**Table 1:** Indicator system under the equity principle.

Dimension name	Indicator name and symbol	Calculation method	Indicator Sources
Historical carbon emission responsibility	Cumulative historical carbon emissions ($HCO_{2o}$)	Cumulative carbon emissions since the baseline	China Energy Statistical Yearbook,China Emission Accounts and Datasets (CEADs)
Transferred carbon emission responsibility	Transferred carbon emissions ($tCO_{2ot}$)	Deviation between actual and reasonable carbon emissions	The data prediction was obtained through calculation using PSO-LSSVM.
Current carbon emission responsibility	Industrial structure ($T_{1}$)	Index of industrial structure rationalization	Theil Index
	Energy consumption structure ($T_{2}$)	Proportion of coal consumption in total energy consumption	China Energy Statistical Yearbook
Population equality	Population size ($T_{3}$)	Resident population	China Statistical Yearbook
Equality of payment capacity	Per capita disposable income ($T_{4}$)	Resident disposable income divided by resident population	Regional Statistical Yearbook, China Statistical Yearbook
Fairness in guarantees	Forest resources ($T_{5}$)	Forest stock volume	China Environmental Statistical Yearbook, Regional Statistical Bulletins
	Crop resources ($T_{6}$)	Actual sown area of crops	China Statistical Yearbook, China Regional Economic Statistical Yearbook, Regional Statistical Yearbooks

The index of industrial structure rationalization, an improved Theil index, effectively measures regional industrial structure rationalization [32]. The calculation formula is given in Eq. (1), where *Y* is output, *L* is employment, *i* represents industry, and *n* is the number of sectors.


$$T_{1}=\sum_{\text{i}=1}^{n}\left(\frac{Y_{i}}{Y}\right)\ln\left(\frac{Y_{i}}{L_{i}}/\frac{Y}{L}\right)$$ (1)

#### 2.1.2. Carbon emission indicators calculation.

We follows the IPCC methodology [33] to calculate carbon emission indicators in Table 1. A province’s carbon emissions are the weighted sum of eight energy types, including coal and coke, using standard coal conversion and CO_2_ emission coefficients. The values are provided in S1 Appendix. Carbon emissions for the o-th province in year t are denoted as $CO_{2ot}$.

Establishing a historical carbon emission baseline is key to equitable quota allocation [34], as it prevents early industrialized regions from shirking responsibility and ensures later-developing regions aren’t overburdened. Existing studies typically use points of similar economic development as baselines. Thus, we group regions based on comparable per capita disposable income to ensure similar economic levels and assign the same baseline for each group, as shown in Table 2.

**Table 2:** Historical carbon emission baseline years for provinces.

Group number	Region	Baseline year
1	Beijing, Guangdong, Jiangsu, Shanghai, Tianjin, Zhejiang	2003
2	Fujian, Hubei, Liaoning, Inner Mongolia, Shandong, Chongqing	2008
3	Anhui, Hainan, Hebei, Hunan, Jilin, Jiangxi	2010
4	Henan, Heilongjiang, Ningxia, Shanxi, Shaanxi, Sichuan	2011
5	Gansu, Guangxi, Guizhou, Qinghai, Xinjiang, Yunnan	2012

The historical cumulative carbon emissions ($HCO_{2o}$) for region o in year t are calculated using Eq. (2), where $t_{0}$ is the baseline year.


$$HCO_{2o}=\sum_{T=t_{0}}^{t}CO_{2ot}$$ (2)

Existing studies often measure inter-regional carbon transfers by the difference between actual and reasonable emissions. Let $mCO_{2ot}$ represent reasonable emissions for region o in year t. Transferred emissions ($tCO_{2ot}$) are calculated using Eq. (3).


$$tCO_{2ot}=CO_{2ot}-mCO_{2ot}$$ (3)

Net carbon emissions are the difference between actual and transferred emissions. Historical net emissions ($T_{7}$) for each region in year t are calculated using Eq. (4).


$$T_{7}=\sum_{T=t_{0}}^{t}\left(CO_{2ot}-tCO_{2ot}\right)$$ (4)

Zhou et al. used economic development, urbanization, openness, population size, industrial structure, energy structure, and technological innovation as independent variables to forecast reasonable carbon emissions, employing Support Vector Machine Regression (SVR) [28]. While SVR performs well, it struggles with complex data distribution and is hyperparameter-dependent. To improve fitting, this study uses the PSO-LSSVM model, which minimizes squared errors and optimizes parameters. Based on Zhou et al.‘s indicators, we forecast reasonable carbon emissions for 30 provincial regions from 2003 to 2022. The independent variable calculations are shown in Table 3.

**Table 3:** Calculation methods for independent variables in the PSO-LSSVM model.

Variable name	Calculation method	Indicator Sources
Economic development level$\left(x_{1}\right)$	Real GDP	China National Statistical Yearbook, Regional Statistical Yearbooks
Population size$\left(x_{2}\right)$	Resident population	Regional Statistical Yearbooks, Regional Statistical Bulletins
Industrial structure$\left(x_{3}\right)$	Index of industrial structure rationalization	Theil Index
Energy consumption structure$\left(x_{4}\right)$	Proportion of coal consumption in total energy consumption	China Energy Statistical Yearbook
Technological innovation$\left(x_{5}\right)$	Proportion of R&D expenditure to GDP	Regional Statistical Yearbooks, Regional Statistical Bulletins
Urbanization level$\left(x_{6}\right)$	Proportion of urban population to total regional population	Regional Statistical Yearbooks, EPS Database
Openness level$\left(x_{7}\right)$	Proportion of actual foreign direct investment utilization to GDP	China National Statistical Yearbook

Let the independent variables in Table 3 be denoted as $x_{i}$and the dependent variable as $CO_{2}$. The sample points for prediction are $\left\{\left(x_{ij},CO_{2j}\right)\text{; i}=1,2,\cdots ,7;j=1,2,\cdots ,600\right\}$. The LSSVM model addresses the following problem:


$$CO_{2}(x)=w^{T}\varphi (x_{j})+b$$ (5)

Here, w is the weight vector, b is the bias, and $e_{j}$ is the error variable. Let γ be the regularization parameter, leading to the optimization problem in Eq. (6).


$$\{\begin{array}{c} \min_{w,b,e}J\left(w,e\right)=\frac{1}{2}w^{T}w+\frac{1}{2}\gamma \sum_{j=1}^{600}e_{j}^{2}\\ s.t.CO_{2j}=w^{T}\varphi (x_{j})+b+e_{j},j=1,2,\cdots 600 \end{array}$$ (6)

By constructing the Lagrangian function and using the radial basis kernel to solve Eq. (6), we obtain Eq. (7), which represents reasonable carbon emissions. Here, $\alpha_{j}$ is the Lagrange multiplier for $x_{j}$, and σ is the width parameter of the radial basis kernel.


$$\hat{CO_{2}(x)}=T_{2}(x)=\sum_{j=1}^{N}\alpha_{j}\exp\left(-\frac{{\left\|x_{j}-x_{k}\right\|}^{2}}{2\sigma^{2}}\right)+b$$ (7)

To improve model performance, the PSO algorithm optimizes parameters γ and σ. Let $V_{i}$ and $X_{i}$ represent the velocity and position of the i-th particle, and $P_{i}$ and $P_{g}$ represent the particle’s best position and the global best, respectively. Velocity and position are updated using Eq. (8), where d is the solution space dimension, k is the iteration number, ω is the inertia weight, and $c_{1}$, $c_{2}$ are acceleration factors. Repeating this process finds the optimal γ and σ.


$$\{\begin{array}{l} V_{id}^{k+1}=\omega (s)V_{\text{id}}^{k}+c_{1}r_{1}\left(P_{id}^{k}-X_{id}^{k}\right)+c_{2}r_{2}(P_{gd}^{k}-X_{gd}^{k})\\ X_{id}^{k+1}=X_{id}^{k}+V_{id}^{k+1} \end{array}$$ (8)

#### 2.1.3. Quota allocation under the principle of equity.

This study constructs a multi-factor equity allocation model using a comprehensive indicator method, which is more scientific than a single indicator approach due to the introduction of weight factors. We use indicators $T_{1}$~$T_{7}$ as equity factors, where $T_{oj}\left(o=1,2,\cdots 30;j=1,2,\cdots 7\right)$ represents the data. After normalization, entropy weights $w_{j}$ are calculated. We establish a multi-factor allocation model based on the equity principle. Let $C_{t}$ represent the total carbon quota for year t, and $T_{oj}$ the average value of the j-th indicator for region o from the baseline year to t. The initial carbon quota for region o in year t ($C_{ot}$) is calculated using Eq. (9).


$$C_{ot}=C_{t}\cdot \left[\sum_{j=1}^{7}\left(\frac{{\bar{T}}_{oj}}{\sum_{o=1}^{30}{\bar{T}}_{oj}}\cdot w_{j}\right)\right]$$ (9)

### 2.2. Efficiency principle considering factor slack

The efficiency principle minimizes resource use and pollution while maintaining equal economic output. It optimizes input-output factors, helping regions achieve carbon reduction targets faster. Regions with high carbon efficiency have greater potential for emission reductions and should receive more quotas. This paper refines the allocation model to enhance resource optimization.

#### 2.2.1. Model improvement and data sources.

Most studies use the ZSG-DEA model for quota allocation under the efficiency principle, but it assumes proportional changes in inputs or outputs and doesn’t account for slack in efficiency. To address this, we adopt the ZSG-SBM model to reallocate the slack of undesired outputs in inefficient DMUs, ensuring all DMUs reach the efficiency frontier and carbon quotas are allocated optimally.

Building on existing research, this paper uses labor, capital, and energy consumption as input factors, GDP as the desired output, and real carbon emissions as the undesired output. Table 4 details the measurement methods and data sources. The perpetual inventory method calculates physical capital stock, where $K_{ot}$ is the current stock, $K_{ot-1}$ is the previous stock, $\delta_{ot}$ is the depreciation rate (9.6% per Zhang et al. [35]), and $I_{ot}$ is the actual total fixed asset formation.

**Table 4:** Indicator system under the efficiency principle.

Indicator name	Calculation method
Labor	Total labor force
Capital	Physical capital stock
Energy consumption	Total energy consumption
GDP	Real GDP
CO_2_ emissions	Actual carbon emissions


$$K_{ot}=K_{ot-1}(1-\delta_{ot})+I_{ot}$$ (10)

#### 2.2.2. Quota allocation under the principle of efficiency.

The quota allocation problem under the efficiency principle adjusts for undesired outputs. We first present the standard efficiency SBM model, output-oriented with undesired outputs. In Eq. (11), o=1,2,⋯N represents the o-th DMU, $\rho^{*}$ is the efficiency score, $x_{io}\left(i=1,2,\cdots ,m\right)$ is the i-th input, $y_{ro}^{g}\left(r=1,2,\cdots ,q\right)$ is the r-th desired output, $b_{ko}^{b}\left(k=1,2,\cdots ,h\right)$ is the k-th undesired output, and $s_{i}^{-},s_{r}^{+},s_{k}^{-}$ are slack variables.


$$\begin{array}{l} \rho^{*}=\min\left[1+\frac{1}{q+h}\left(\sum_{r=1}^{q}\frac{s_{r}^{+}}{y_{ro}}+\sum_{k=1}^{h}\frac{s_{k}^{-}}{b_{ko}}\right)\right]\\ s.t. \{\begin{array}{c} x_{io}=\sum_{j=1}^{n}\lambda_{j}x_{ij}+s_{i}^{-},i=1,2,\cdots ,m\\ \begin{array}{l} y_{ro}^{g}=\sum_{j=1}^{n}\lambda_{j}y_{rj}-s_{r}^{+},r=1,2,\cdots ,q\\ b_{ko}^{b}=\sum_{j=1}^{n}\lambda_{j}b_{kj}+s_{k}^{-},k=1,2,\cdots ,h \end{array}\\ \sum_{j=1}^{n}\lambda_{j}=1\\ s_{i}^{-},s_{r}^{+},s_{k}^{-},\lambda_{j}\geq 0 \end{array} \end{array}$$ (11)

The ZSG-SBM model is built on Eq. (11), following Wang and Kong [21], as shown in Eq. (12).


$$\begin{array}{l} \rho^{*}=\min{\left(\frac{b_{ko}^{b}-s_{k}^{-}}{b_{ko}^{b}}\right)}^{ZSG}\\ s.t. \{\begin{array}{c} x_{io}=\sum_{j=1}^{n}\lambda_{j}x_{ij}+s_{i}^{-},i=1,2,\cdots ,m\\ \begin{array}{l} y_{ro}^{g}=\sum_{j=1}^{n}\lambda_{j}y_{rj}-s_{r}^{+},r=1,2,\cdots ,q\\ b_{ko}^{b}{\left(\frac{b_{ko}-s_{k}^{-}}{b_{ko}}\right)}^{ZSG}\text{=}\sum_{j=1}^{n}\lambda_{j}b_{kj},k=1,2,\cdots ,h \end{array}\\ \sum_{j=1}^{n}\lambda_{j}=1\\ s_{i}^{-},s_{r}^{+},s_{k}^{-},\lambda_{j}\geq 0 \end{array} \end{array}$$ (12)

Let the carbon emission efficiency of region $p\left(p=1,2,\cdots ,N\right)$ be $\rho_{k}^{p}$, and the initial carbon quota be $b_{kp}^{b}$. To improve efficiency, region p must reduce emissions by $b_{kp}^{b}\left(1-\rho_{k}^{p}\right)$ units. Conversely, other regions $o\left(o\neq p\right)$ can increase their emissions by $b_{kp}^{b}\left(1-\rho_{k}^{p}\right)\left(b_{ko}^{b}/\overset{N}{\underset{o=1,o\neq p}{\sum }}b_{ko}^{b}\right)$ units. The adjusted carbon quota for region o is given by Eq. (13). After multiple iterations, all DMUs reach optimal efficiency, and the resulting quota reflects allocation under the efficiency principle. Initial quotas for each province are estimated by the region’s average annual carbon emissions as a ratio of the national total.


$$b_{ko}^{\mathrm{b\prime}}=b_{ko}^{b}+\sum_{o=1,o\neq p}^{N}\left[b_{kp}^{b}(1-\rho_{k}^{p})\frac{b_{ko}^{b}}{\sum_{o=1,o\neq p}^{N}b_{ko}^{b}}\right]-b_{ko}^{b}(1-\rho_{k}^{o})$$ (13)

### 2.3. Comprehensive distribution principle based on local conditions

While national carbon targets are set top-down, a bottom-up approach that considers provincial realities is essential. By weighing regional abatement costs, the quota allocation becomes more rational and feasible. This paper introduces the AMLC principle, a two-stage process: first, applying the equity principle to allocate total quotas by region category; second, differentiating within each category based on the economic benefits of carbon reduction. This phased approach balances equity and efficiency, enabling flexible policy adjustments in a changing economic environment.

#### 2.3.1. Classification of Chinese provinces based on hierarchical clustering.

Green and Stern noted a structural shift in China’s emission reduction landscape post-2013 due to economic development goals [36]. Historical and current carbon responsibility, population equality, payment capacity, and guarantee fairness capture regional disparities in carbon reduction and economic development. To accurately reflect each region’s development, we classify provincial regions using $T_{1}\sim T_{7}$ indicator data from 2014 to 2022.

This study chooses hierarchical clustering for its intuitive representation of clustering hierarchy and relationships. We use the mean values ${\bar{T}}_{i}$ of indicators $T_{j}\left(j=1,2,\cdots ,7\right)$ from 2014 to 2022 as clustering features. Euclidean distance is calculated using Eq. (14) for the dataset $\left\{{\bar{T}}_{1j},{\bar{T}}_{2j},\cdots {\bar{T}}_{30j}\right\}$, where $\bar{T_{mj}}$ and $\bar{T_{nj}}$ represent the values of samples $\bar{T_{m}}\left(m=1,2,\cdots 30\right)$ and $\bar{T_{n}}\left(n=1,2,\cdots 30\right)$ for the jth feature.


$$d\left({\bar{T}}_{m},{\bar{T}}_{n}\right)=\sqrt{\sum_{j=1}^{7}{\left({\bar{T}}_{mj}-{\bar{T}}_{nj}\right)}^{2}}$$ (14)

Each sample is treated as an individual cluster, and the Ward method is used to measure intra-cluster distance, progressively merging the most similar clusters. As shown in Eq. (15), $W_{m}$ and $W_{n}$ represent the clusters to be merged. The distance matrix is updated after each merge, repeating until the stopping condition is met. With 30 samples, we set the number of clusters to k=6 for interpretability, denoting the clusters as $CR_{k}$ $\left(k=1,2,\cdots ,6\right)$.


$$Ward'sdistance=\frac{{\left\|W_{m}\cup W_{n}\right\|}^{2}-{\left\|W_{m}\right\|}^{2}-{\left\|W_{n}\right\|}^{2}}{\left|W_{m}\cup W_{n}\right|}$$ (15)

#### 2.3.2. The two-stage allocation under the AMLC principle.

***Stage 1: Allocation Among Clusters***

Unlike equity-based allocation, this study uses the CRITIC method to determine indicator weights, considering both dispersion and correlation for more robust results. Based on standardized data $T_{oj}^{\text{'}}$ $\left(o=1,2,\cdots 30\right)$ from 2014 to 2022, the standard deviation $\sigma_{j}$ and correlation coefficient $r_{jl}\left(l=1,2,\cdots ,7\right)$ between each pair of indicators were calculated. The information content $I_{j}$ for each indicator is derived using Eq. (16), and the weight $w_{j}$ is the ratio of its information content to the total across all indicators.


$$I_{j}=\sigma_{j}\times \left(1-\frac{1}{6}\sum_{l=1}^{7}\left|r_{jl}\right|\right)$$ (16)

We calculate the centroid values for each cluster group $CR_{k}$ $\left(k=1,2,\cdots ,6\right)$ for indicator $T_{j}$ using provincial data. The centroid value ${\dot{G}}_{ki}$for each indicator $T_{i}$ in cluster $CR_{k}$ is calculated using Eq. (17), where ${\dot{t}}_{ei}$ is the dimensionless data, and $n_{k}$ is the number of provinces in the k-th cluster.


$${\dot{G}}_{ki=}\frac{\sum_{e\in k}{\dot{t}}_{ei}}{n_{k}},k=1,2,\cdots 6;i=1,2,\cdots 7$$ (17)

The carbon emission quota allocation ratio for each cluster $CR_{k}$ is obtained by multiplying the weight $w_{j}$ of each indicator by the group centroid and summing across all seven indicators. This ratio is then multiplied by the total quota $C_{t}$ to determine the allocation for each group in year t.

***Stage 2: Quota Allocation Within Each Cluster Group***

Reducing carbon emissions increases socioeconomic costs, and quota allocation inherently restricts economic development. Within each cluster, distribution is based on the marginal abatement cost (SPC). A higher SPC indicates greater GDP loss per unit of carbon reduced. To maximize economic benefits, quotas should prioritize regions with higher SPC. Using shadow prices to measure SPC, this study modifies the SBM-DEA model via dual transformation to derive a profit-maximization function. The marginal abatement cost of CO₂, $spc_{o}\left(o=1,2,\cdots 30\right)$, is calculated, where $p_{ro}$, $p_{io}$, and $p_{ko}$ represent the shadow prices of desirable outputs, inputs, and undesirable outputs.


$$\begin{array}{l} \max A=\sum_{r=1}^{q}p_{ro}y_{ro}^{g}-\sum_{i=1}^{m}p_{io}x_{io}-\sum_{k=1}^{h}p_{ko}b_{ko}^{b}\\ s.t. \{\begin{array}{c} A\leq 0\\ p_{ro}\geq \frac{1+A}{(q+h)y_{ro}^{g}}\\ p_{io}\geq \frac{1}{mx_{io}}\\ p_{ko}\geq \frac{1+A}{(q+h)b_{ko}^{b}} \end{array} \end{array}$$ (18)

Following Wang and Kong [21] and Duan et al. [37], $spc_{o}$ is expressed in Eq. (19), capturing the relationship between GDP and carbon emissions. Here, $r_{yo}$ represents the market price of economic output, typically set at 1 yuan. Thus, $spc_{o}$ is calculated as the ratio of the shadow price of undesirable output to that of desirable output, i.e., $p_{ko}$/$p_{ro}$.


$$spc_{o}=r_{yo}\times \frac{\partial {\vec{D}}_{0}\left(x,y,b,g_{y},g_{ca}\right)/\partial b}{\partial {\vec{D}}_{0}\left(x,y,b,g_{y},g_{ca}\right)/\partial y}=r_{yo}\times \frac{p_{ko}}{p_{ro}}=\frac{p_{ko}}{p_{ro}}$$ (19)

The allocation weight for province o in cluster $CR_{k}$ is defined as the ratio of its marginal abatement cost to the total costs of all provinces in the cluster. Multiplying this weight by the total cluster quota determines the final quota for the province.

## 3. Analysis of spatial differences in regional development in China

In the previous section, we proposed a tailored quota allocation scheme based on the AMLC principle, starting with categorizing the development status of 30 Chinese provinces and municipalities. Here, we visualize the spatiotemporal evolution of carbon emissions transfers and analyze regional development differences using hierarchical clustering results.

### 3.1. Evolution of regional carbon emission transfers

Differences in production factor prices and industrial divisions among regions drive the transfer of carbon emissions. This refers to the transfer of carbon emissions between regions, often via products and services [38]. There is a complex network of carbon transfers between provinces in China. Analyzing the flow of carbon transfers reveals low-carbon development opportunities in each region, essential for sustainable development. A positive value for transferred carbon emissions indicates that CO_2_ has been transferred to other regions, making it a “carbon output” area; conversely, it would be called a “carbon input” area. Since 2013 marked a turning point in China’s emission reduction efforts, we compared carbon transfer levels across provincial regions from 2003–2013 and 2014–2022.

Data from S2 Appendix indicate that from 2014 to 2022, the national averages for actual GDP, industrial structure rationalization index, energy consumption index, R&D intensity, and urbanization index across provinces were 19,955.77 (billion yuan), 0.17, 0.36, 0.02, and 0.62, respectively, all higher than the 2003–2013 levels. This reflects the optimization of China’s economic structure and a gradual shift toward high-quality development. The rise in R&D investment and urbanization rates indicates enhanced regional competitiveness. The openness index dropped from 0.34 to 0.25, reflecting China’s focus on a “dual circulation” economy amid global challenges such as Sino-US trade tensions and the COVID-19 pandemic.

Against this backdrop, carbon transfer patterns among provinces have shifted. In terms of numbers, from 2014 to 2022, the number of carbon-exporting regions decreased from 17 to 15, while carbon-importing regions increased from 13 to 15, concentrating carbon emission pressures in fewer areas. Shandong, the largest carbon exporter, transferred an annual average of 50.58 million tons of CO_2_ equivalent in standard coal, while Jilin, the largest importer, received 53.36 million tons annually. The gap in transferred emissions widened from 99.19 to 103.95 million tons, exacerbating regional imbalances.

From the perspective of transfer trends, from 2003 to 2013, Heilongjiang, Guangxi, and Shaanxi were major carbon exporters, with annual transfers of 17.08, 16.03, and 12.55 million tons of standard coal, respectively. These regions had GDPs below the national average (8,788.28 billion yuan) and higher-than-average industrial structure rationalization indices (0.278). Shanxi and Hebei also exported 17.01 and 5.95 million tons annually, with energy consumption indices far exceeding the national average (0.480), indicating a reliance on energy-intensive, low-value-added industries. In contrast, Chongqing, Jilin, and Hunan were carbon importers, with annual transfers of −80.36, −31.68, and −18.34 million tons, respectively, due to limited fossil fuel reserves. Thus, from 2003 to 2013, carbon flows moved from energy-rich, market-dependent regions to energy-poor regions with limited fossil fuel reserves. From 2014 to 2022, Zhejiang, Hunan, Tianjin, and Beijing shifted from carbon importers to exporters, transferring 40.47, 15.91, 11.46, and 5.34 million tons annually. Conversely, Hubei, Hebei, and Gansu transitioned from exporters to importers, with annual transfers of −34.02, −16.31, and −11.82 million tons, respectively. During this period, developed regions shifted high-energy industries to less developed areas, creating a new pattern of carbon emission transfers to these strategic locations.

### 3.2. Regional attribute classification and feature analysis

In the previous section, we detailed China’s carbon emission transfer paths over two periods, highlighting imbalances in economic development and resource use. A unified low-carbon policy may not address regional needs, making it crucial to define regional endowment categories. Using 2014–2022 data and the classification method in Section 2.3.1, this section categorizes China’s 30 provincial regions into six groups, as shown in Table 5.

**Table 5:** The classification results of China’s 30 provincial-level administrative regions.

Type	Name of province	Number
1	Beijing, Shanghai	2
2	Fujian, Hainan, Qinghai, Tianjin, Zhejiang	5
3	Gansu, Guangxi, Guizhou, Jilin, Ningxia, Shanxi, Shaanxi, Xinjiang	8
4	Anhui, Hebei, Henan, Hubei, Hunan, Jiangxi, Liaoning, Chongqing	8
5	Heilongjiang, Inner Mongolia, Sichuan, Yunnan	4
6	Guangdong, Jiangsu, Shandong	3

**Fig 2:**
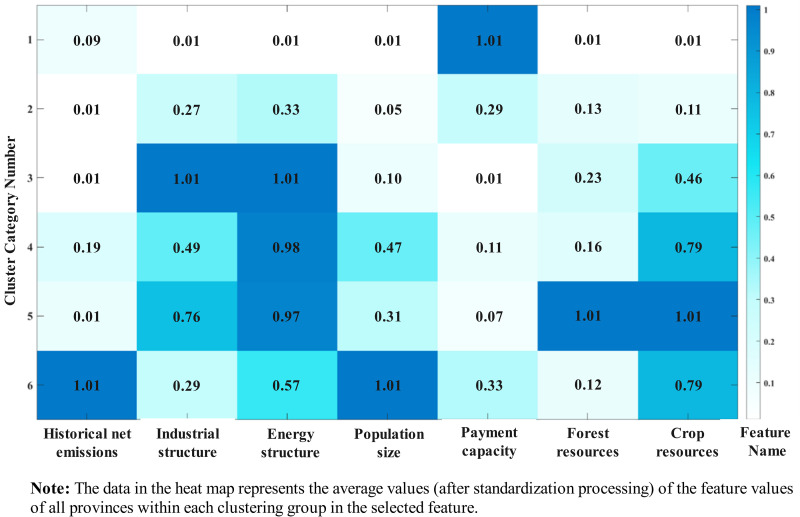
Heatmap of mean characteristics by category.

This study standardizes the feature values and presents the average performance of each cluster across different characteristics using a heatmap (Fig 2). The first cluster, consisting of Beijing and Shanghai, has the highest mean value for per capita disposable income at 1.01. However, its average performance on the industrial structure rationalization index, energy consumption index, forest stock volume, and crop sown area, all at 0.01, is significantly lower than the other clusters. This indicates that while this group has the best economic conditions and highly rational industrial and energy structures, it suffers from a severe shortage of agricultural and forestry resources. The second cluster, which includes Fujian, Hainan, Qinghai, Tianjin, and Zhejiang, has a low carbon emission responsibility, with a mean cumulative net carbon emission value of 0.01. Its industrial structure rationalization index (0.27) and energy consumption index (0.33) are slightly higher than those of the first cluster, indicating relatively balanced industrial and energy structures. The third cluster, which includes Gansu, Guangxi, and others, has the most inefficient industrial structure and the lowest per capita disposable income, with mean values of 1.01 and 0.01, respectively.

Compared to the first three clusters, the last three clusters have higher historical carbon emissions and are predominantly carbon-exporting regions. They perform well in terms of crop sown area (mean values of 0.79, 1.01, and 0.79) and permanent population (mean values of 0.47, 0.31, and 1.01), reflecting densely populated agricultural provinces. Despite their similarities, these regions also exhibit notable differences: the fourth cluster, including Anhui and Hebei, has a relatively poor energy consumption structure (mean value of 0.98), only slightly better than that of the third cluster. The fifth cluster, which includes Heilongjiang, Inner Mongolia, and others, has abundant carbon sink resources, with the highest mean forest stock volume nationwide at 1.01. The sixth cluster, comprising Guangdong, Jiangsu, and Shandong, has the highest historical cumulative net carbon emissions and permanent population, both with mean values of 1.01, indicating the largest carbon emission responsibility and population size. This analysis provides valuable insights for developing quota allocation schemes based on regional classification.

## 4. Allocation of quotas and selection of schemes under the “peak carbon” scenario

### 4.1. Estimation of national carbon emissions in 2030

The “Action Plan for Peak Carbon before 2030” sets a target where by 2030, carbon dioxide emissions per unit of GDP will decrease by over 65% compared to 2005. Tian and Lin calculated the annual average change rate of carbon emission intensity using historical data to estimate future years’ intensity levels, assuming GDP growth at an annual rate of 5% to 6% [39]. However, China’s economic focus has shifted towards quality efficiency rather than speed and scale [40]. There is room for improvement in determining values for changes in carbon emission intensity and GDP growth rates annually. Resetting parameters based on existing research is necessary and feasible.

The Chinese government’s Development Research Center predicts that China’s annual GDP growth rate will be between 4% and 6% from 2020 to 2030. This study discusses the social and economic development assumptions made by Zhang et al. [41], which suggest that GDP growth rates will be 5.8% during the “14th Five-Year Plan” period (2021~2025) and 4.8% during the “15th Five-Year Plan” period (2026~2030), with annual carbon intensity reduction rates of 4.5% and 5%. The formula for calculating carbon emissions quota quantity in year t is shown as Eq. (20).


$$C_{\text{t}}= \{\begin{array}{l} GDP_{2022}\times {\left[1+5.8\%\right]}^{t-2022}\times I_{2022}\times {\left(1-4.5\%\right)}^{t-2022},t=2023,2024,2025\\ GDP_{2025}\times {\left[1+4.8\%\right]}^{t-2025}\times I_{2025}\times {\left(1-5.0\%\right)}^{t-2025},t=2026,2027,\cdots 2030 \end{array}$$ (20)

Among them, $GDP_{t}$ and $I_{t}$ represent the gross domestic product and carbon intensity of year t. By summing up the annual carbon quota quantity, we can obtain the total quota for 2023~2030 as C. The total GDP in 2022 is 12.01 trillion yuan, with carbon dioxide emissions amounting to 13.08 billion tons of standard coal. Substituting this data into the equation will give us an estimate of annual quotas for 2023~2030, shown in S3 Appendix . In this scenario, China’s carbon emissions are expected to peak at 13.49 billion tons of standard coal in 2025 before gradually decreasing to reach 13.20 billion tons by 2030. This result is consistent with the conclusion drawn by Wang et al. [42], who predicted through Monte Carlo simulation that China’s overall carbon emissions will peak between 2021 and 2025, with a peak value ranging from 13 to 16 billion tons of carbon dioxide.

### 4.2. Analysis of quota allocation and carbon emission space balance under three principles

Due to space constraints, we cannot allocate total quotas for each year in S3 Appendix. Using 2025 as a case study, this research examines the pros and cons of quota allocation based on equity, efficiency, and AMLC principles using data from 30 provincial-level administrative regions in China. We choose a distribution plan that maximizes profits for the majority of provinces.

#### 4.2.1. Quota allocation results based on the principle of equity.

As is shown in Table 6, under the equity principle, the weights calculated by the entropy weight method show that forest stock volume and crop sown area have the highest weights, at 44.42% and 20.75%, respectively, exerting the greatest influence on the allocation results. Next is permanent population, with a weight of 14.91%. The remaining factors—energy consumption index (8.82%), per capita disposable income (6.41%), industrial structure rationalization index (2.47%), and cumulative net carbon emissions (2.22%)—all have weights below 10%, indicating a smaller impact on the allocation results. Based on this, regions rich in agricultural and forestry resources, such as Sichuan (113,292.71 million tons), Heilongjiang (112,503.66 million tons), and Yunnan (108,946.09 million tons), receive the highest quotas. Quotas are also skewed toward populous provinces such as Henan (55,450.24 million tons), Guangdong (52,140.17 million tons), and Shandong (47,924.70 million tons). Regions with lower quotas generally lack forestry resources and large populations, such as Qinghai (10,234.58 million tons), Tianjin (10,806.57 million tons), and Beijing (11,569.35 million tons).

**Table 6:** 2025 Quota allocation results under the equity principle.

Region	Cluster type	Quota amount (10 ktce)	Quota ranking	Carbon emissions (10 ktce)	Carbon emissions ranking	Quota surplus (10 ktce)	Quota surplus ranking
Sichuan	5	113292.71	1	27500.87	19	85791.84	1
Yunnan	5	108946.09	3	27269.33	20	81676.76	2
Heilongjiang	5	112503.66	2	32784.02	16	79719.64	3
Jilin	3	64455.87	5	17829.74	25	46626.13	4
Guangxi	3	52387.23	7	30871.20	17	21516.03	5
Jiangxi	4	44631.61	13	23257.40	23	21374.21	6
Hunan	4	49539.83	9	29462.07	18	20077.76	7
Guizhou	3	39855.69	17	25405.97	21	14449.72	8
Chongqing	4	25795.11	24	12159.14	27	13635.97	9
Fujian	2	46050.53	11	35979.91	15	10070.61	10
Henan	4	55450.24	6	46515.03	11	8935.21	11
Hubei	4	45609.93	12	38600.00	13	7009.94	12
Gansu	3	28994.43	22	22977.70	24	6016.73	13
Qinghai	2	10234.58	30	5107.28	30	5127.30	14
Hainan	2	12286.15	27	7395.64	28	4890.51	15
Beijing	1	11569.35	28	7035.86	29	4533.49	16
Anhui	4	41370.90	16	40796.75	12	574.15	17
Tianjin	2	10806.57	29	17605.00	26	−6798.43	18
Shaanxi	3	42345.94	14	50847.25	10	−8501.31	19
Shanghai	1	13652.47	26	25209.46	22	−11556.99	20
Inner Mongolia	5	92978.35	4	105628.91	3	−12650.56	21
Ningxia	3	13843.89	25	37080.54	14	−23236.64	22
Guangdong	6	52140.17	8	78902.12	6	−26761.96	23
Xinjiang	3	37472.95	19	69371.23	9	−31898.28	24
Hebei	4	41659.50	15	75912.31	7	−34252.81	25
Zhejiang	2	31659.27	21	69391.97	8	−37732.71	26
Jiangsu	6	38199.14	18	82420.63	5	−44221.49	27
Liaoning	4	35443.36	20	93272.36	4	−57829.00	28
Shanxi	3	27895.12	23	113439.50	2	−85544.38	29
Shandong	6	47924.70	10	153616.36	1	−105691.66	30

Note: “ktce” stands for kilotons of coal equivalent.

By subtracting actual carbon emissions in 2025 from the allocated quotas, we can determine the surplus or deficit of carbon emissions in each region. Under the equity principle, 17 regions show a carbon surplus, mainly concentrated in Southwest, Central-South, and Northeast China. These surpluses can be categorized as follows: Sichuan, Heilongjiang, Fujian, Gansu, Qinghai, Hainan, and Beijing, which have rational energy consumption structures with low energy use, ensuring low carbon emissions and resulting in surplus quotas; Yunnan, Jilin, Guangxi, and Guizhou, where relatively small economic size limits carbon emissions growth despite suboptimal energy consumption structures; and Jiangxi, Hunan, Chongqing, Henan, Hubei, and Anhui, which belong primarily to the fourth cluster with poor energy consumption structures but receive sufficient quotas due to their large crop sown areas, enough to cover their carbon emissions during development. In contrast, 13 regions in North, East, and Northwest China show a quota deficit: Tianjin and Shanghai, with limited agricultural and forestry resources and large populations, face deficits due to insufficient quotas, while regions like Shandong, Shanxi, Liaoning, and Jiangsu, with robust energy industries and strong industrial productivity, experience deficits due to high carbon emissions. Lastly, of the six clusters identified, five achieve a quota surplus under the equity principle, but the sixth cluster, consisting of Guangdong, Jiangsu, and Shandong, faces a deficit, indicating that the equity principle still requires improvement to ensure balanced benefits across regions with varying resource endowments.

#### 4.2.2. Quota allocation results under the efficiency principle.

The results of quota allocation based on the efficiency principle are calculated using the ZSG-SBM model. Detailed results can be found in Table 7. Initial efficiency values show that Jiangsu, Guangdong, Zhejiang, Fujian, Shanghai, Chongqing, Beijing and 11 other provinces and cities have an efficiency value of 1. This indicates they have reached the effective frontier in terms of energy utilization efficiency and environmental regulation intensity. These areas can control carbon emissions effectively while ensuring economic development. Other regions have not yet reached this level of efficiency. To address this issue, we made four iterations adjustments while keeping total carbon emissions constant to determine quotas under the efficiency principle when all provinces’ efficiencies are at 1. Jiangsu Province has the highest quota at 187606.57 million tons of standard coal followed by Guangdong (179597.71 million tons), Zhejiang (157950.62 million tons) and Ningxia (84403.05 million tons); Qinghai has the lowest quota amount at 5107.28 million tons followed by Jilin (17829.74 million tons), Gansu (22977.70 million tons) and Xinjiang (69371.23 million tons).

**Table 7:** 2025 quota allocation results under the efficiency principle.

Region	Cluster type	Initial		1st Iteration	2nd Iteration	3rd Iteration	4th Iteration	Result	
Carbon emissions (10 ktce)	Efficiency value	Efficiency value	Efficiency value	Efficiency value	Efficiency value	Carbon quota (10 ktce)	Quota surplus (10 ktce)
Jiangsu	6	82420.63	1.00	1.00	1.00	1.00	1.00	187606.57	105185.93
Guangdong	6	78902.12	1.00	1.00	1.00	1.00	1.00	179597.71	100695.58
Zhejiang	2	69391.97	1.00	1.00	1.00	1.00	1.00	157950.62	88558.64
Ningxia	3	37080.54	1.00	1.00	1.00	1.00	1.00	84403.05	47322.52
Fujian	2	35979.91	1.00	1.00	1.00	1.00	1.00	81897.79	45917.88
Hunan	4	29462.07	1.00	1.00	1.00	1.00	1.00	67061.83	37599.76
Shanghai	1	25209.46	1.00	1.00	1.00	1.00	1.00	57382.00	32172.54
Sichuan	5	27500.87	0.76	0.90	0.97	1.00	1.00	47329.36	19828.49
Chongqing	4	12159.14	1.00	1.00	1.00	1.00	1.00	27676.74	15517.60
Henan	4	46515.03	0.57	0.79	0.94	1.00	1.00	59484.24	12969.21
Anhui	4	40796.75	0.57	0.78	0.94	1.00	1.00	51909.08	11112.33
Hainan	2	7395.64	1.00	1.00	1.00	1.00	1.00	16834.02	9438.39
Beijing	1	7035.86	1.00	1.00	1.00	1.00	1.00	16015.09	8979.23
Tianjin	2	17605.00	0.62	0.81	0.95	1.00	1.00	24513.05	6908.06
Qinghai	2	5107.28	1.00	1.00	1.00	1.00	1.00	11625.24	6517.95
Hubei	4	38600.00	0.39	0.64	0.89	0.99	1.00	33902.28	−4697.72
Jilin	3	17829.74	0.32	0.56	0.85	0.99	1.00	12787.05	−5042.69
Jiangxi	4	23257.40	0.29	0.53	0.83	0.98	1.00	15115.58	−8141.81
Gansu	3	22977.70	0.25	0.48	0.80	0.98	1.00	12805.20	−10172.50
Guizhou	3	25405.97	0.23	0.46	0.78	0.98	1.00	13280.40	−12125.57
Yunnan	5	27269.33	0.24	0.46	0.79	0.98	1.00	14462.86	−12806.47
Guangxi	3	30871.20	0.20	0.42	0.76	0.98	1.00	14075.63	−16795.57
Heilongjiang	5	32784.02	0.21	0.42	0.76	0.98	1.00	15211.72	−17572.31
Shaanxi	3	50847.25	0.14	0.31	0.63	0.96	1.00	14419.53	−36427.72
Hebei	4	75912.31	0.14	0.32	0.68	0.97	1.00	22764.67	−53147.64
Shandong	6	153616.36	0.31	0.58	0.87	0.99	1.00	100310.63	−53305.73
Xinjiang	3	69371.23	0.09	0.21	0.55	0.94	1.00	13182.92	−56188.31
Liaoning	4	93272.36	0.10	0.25	0.61	0.96	1.00	20492.76	−72779.60
Inner Mongolia	5	105628.91	0.07	0.18	0.48	0.93	1.00	14341.44	−91287.47
Shanxi	3	113439.50	0.06	0.17	0.49	0.93	1.00	15206.50	−98233.00

Note: “ktce” stands for kilotons of coal equivalent; The efficiency values are those measured by the ZSG-SBM model at each iteration, representing the proximity of the decision-making unit (DMU) to the efficiency frontier.

Under the efficiency principle, only 15 regions have surplus quotas, which is fewer than under the principle of equity. When comparing initial and final data, it is evident that regions like Jiangsu and Guangdong, which were already at the efficient frontier initially, saw significant increases in their carbon quota amounts after iterations. Conversely, provinces like Sichuan and Henan, where efficiency was initially less than 1, experienced reductions in available carbon quotas despite efficiency improvements. Therefore, under the efficiency principle adjustment mechanism: high-efficiency regions increase their carbon emissions to enhance efficiency while low-efficiency regions decrease theirs. These two types of regions engage in a game under total control principles to ultimately achieve optimal distribution. While this mechanism ensures relative efficiency improvement among regions, it also widens the gap between regional quotas further. High-efficiency areas can utilize surplus carbon quotas to bolster their development advantages while low-efficiency areas will encounter resource shortages and stringent restrictions on economic activities. In conclusion, distributing resources based on an efficiency principle will exacerbate regional imbalances in China’s development and impact the achievement of sustainable development goals.

#### 4.2.3. Allocation results under the AMLC principle.

Under the AMLC principle, forest stock volume has the highest weight (26.40%), followed by crop sown area (17.68%), population size (17.11%), energy consumption index (12.82%), and per capita disposable income (11.93%). The industrial structure rationalization index (7.16%) and cumulative net carbon emissions (6.91%) have the lowest weights. Compared to the entropy weight method, the CRITIC method lessens the dominance of equity indicators in the allocation, resulting in a more balanced distribution. Using Eq. (16), the index weights are multiplied by the corresponding cluster center proportions to obtain the quota weights for classes 1–6 (Table 8). More than half of the quota resources are allocated to classes 5 and 6, which typically have unsustainable energy consumption patterns, high historical carbon emissions, large populations, and abundant agricultural and forestry resources. This suggests that the AMLC principle’s first-stage allocation not only considers current resource endowments and economic conditions but also compensates regions with high carbon emission responsibilities, easing the conflict between emission reduction obligations and historical carbon emissions. The second-stage allocation leverages differences in shadow prices of carbon emissions within the clusters (SPC, representing marginal abatement costs). Regions with higher shadow prices are allocated more quotas. Comparing provincial data based on SPC, it’s clear that economically developed regions generally have higher shadow prices than underdeveloped areas. Thus, the second-stage allocation ensures resources are directed to regions where efficient emission reductions are most likely.

**Table 8:** 2025 Quota allocation results under the AMLC principle.

Cluster type	Quota weight for each category	Region	Proportion of each province calculated by SPC (%)	Quota allocation (10 ktce)	Quota allocation ranking	Actual carbon emissions (10 ktce)	Quota surplus (10 ktce)	Quota surplus ranking
1	7.48%	Beijing	73.89%	74527.85	5	7035.86	67491.99	4
		Shanghai	26.11%	26332.48	17	25209.46	1123.02	16
2	6.90%	Fujian	28.99%	26978.90	16	35979.91	−9001.02	18
		Zhejiang	23.30%	21682.59	22	69391.97	−47709.39	24
		Tianjin	19.21%	17882.74	23	17605.00	277.74	17
		Hainan	16.04%	14930.73	24	7395.64	7535.10	10
		Qinghai	12.46%	11595.07	26	5107.28	6487.79	13
3	13.16%	Guangxi	20.57%	36516.25	10	30871.20	5645.05	14
		Jilin	19.57%	34736.12	11	17829.74	16906.39	8
		Guizhou	18.05%	32030.65	12	25405.97	6624.67	12
		Gansu	14.13%	25088.64	19	22977.70	2110.94	15
		Shaanxi	13.99%	24834.40	20	50847.25	−26012.85	22
		Xinjiang	6.30%	11177.40	27	69371.23	−58193.83	25
		Shanxi	4.62%	8200.86	28	113439.50	−105238.64	30
		Ningxia	2.77%	4916.91	30	37080.54	−32163.63	23
4	17.16%	Chongqing	25.82%	59773.40	7	12159.14	47614.26	6
		Hunan	16.15%	37384.52	9	29462.07	7922.45	9
		Henan	13.37%	30957.49	13	46515.03	−15557.54	20
		Jiangxi	12.92%	29895.69	14	23257.40	6638.29	11
		Hubei	12.27%	28394.13	15	38600.00	−10205.87	19
		Anhui	10.62%	24589.92	21	40796.75	−16206.83	21
		Hebei	5.39%	12466.88	25	75912.31	−63445.43	26
		Liaoning	3.46%	8003.15	29	93272.36	−85269.21	28
5	30.47%	Sichuan	52.26%	214810.16	1	27500.87	187309.29	1
		Yunnan	24.58%	101006.72	4	27269.33	73737.39	3
		Heilongjiang	17.05%	70081.01	6	32784.02	37296.98	7
		Inner Mongolia	6.11%	25109.76	18	105628.91	−80519.16	27
6	24.84%	Guangdong	45.74%	153286.41	2	78902.12	74384.29	2
		Jiangsu	38.96%	130542.85	3	82420.63	48122.22	5
		Shandong	15.30%	51261.68	8	153616.36	−102354.68	29

Note: “ktce” stands for kilotons of coal equivalent.

Among the top ten provinces in terms of quota allocation, 60% belong to classes 5 and 6. Sichuan (2,148.16 million tons), Guangdong (1,532.86 million tons), and Jiangsu (1,305.42 million tons) received the highest quotas, confirming the compensatory effect of the allocation plan on regions with high carbon emission responsibilities. Regions in classes 1, 3, and 4 have varying levels of economic development and energy consumption structures, but all share the commonality of low carbon emission responsibilities, and generally received quotas above the national average. This shows that the AMLC principle ensures a more balanced distribution of quotas, rather than being biased towards a specific region type. Due to the low average values of the indicators in class 2, this group received the lowest quotas overall.

Meanwhile, under this principle, 17 provinces and cities have a positive quota surplus, which matches the number under the equity principle, but the regions covered are more comprehensive, spanning classes 1–6. Additionally, Shanxi (−105,238.64 million tons), Shandong (−102,354.68 million tons), Liaoning (−85,269.21 million tons), Inner Mongolia (−80,519.16 million tons), and Hebei (−63,445.43 million tons) have significant quota deficits. These regions play crucial roles in national energy supply and heavy industrial development, leading to some of the highest carbon emissions in the country, far exceeding their allocated quotas. This indicates that even an optimized allocation plan cannot fully mitigate the emission reduction pressure faced by high-carbon regions, highlighting the importance of industrial structure upgrades and technological innovation.

### 4.3. Selection of distribution principles based on global malmquist-luenberger index

#### 4.3.1. Calculation method.

The study thoroughly examined the pros and cons of equity, efficiency, and AMLC distribution principles, concluding that the efficiency principle is not aligned with China’s sustainable development goals. Under the equity and AMLC principles, 17 provinces and cities achieved quota surpluses. While the advantages of the AMLC principle for rational quota distribution were emphasized, our research is limited to data up to 2025. To monitor the long-term performance of the two quota principles, we apply the Global Malmquist-Luenberger (GML) index, based on the DEA model, to measure green productivity from 2023 to 2030. Using the global directional distance function $D^{G}\left(x^{t},y^{t},b^{t}\right)$ and the global production possibility set, the GML productivity index from period t to t+1 is defined. This index reflects changes in green productivity between consecutive periods, where a value greater than 1 indicates increased productivity, and a value less than 1 indicates a decrease. The GML index is further broken down into Efficiency Change ($EC^{t,t+1}$), indicating changes in green technical efficiency, and Technological Change ($TC^{t,t+1}$), indicating technological progress. Specifically, $EC^{t,t+1}>1(EC^{t,t+1}<1)$ represents an improvement (decline) in green technical efficiency, and $TC^{t,t+1}>1(TC^{t,t+1}<1)$ indicates technological progress (regression).


$$\begin{array}{l} GML^{t,t+1}\left(x^{t},y^{t},b^{t},x^{t+1},y^{t+1},b^{t+1}\right)=\frac{1+D^{G}\left(x^{t},y^{t},b^{t}\right)}{1+D^{G}\left(x^{t+1},y^{t+1},b^{t+1}\right)}\\ =\frac{1+D^{t}\left(x^{t},y^{t},b^{t}\right)}{1+D^{t+1}\left(x^{t+1},y^{t+1},b^{t+1}\right)}\times \left[\frac{1+D^{G}\left(x^{t},y^{t},b^{t}\right)}{1+D^{t}\left(x^{t},y^{t},b^{t}\right)}\times \frac{1+D^{t+1}\left(x^{t+1},y^{t+1},b^{t+1}\right)}{1+D^{G}\left(x^{t+1},y^{t+1},b^{t+1}\right)}\right]\\ =EC^{t,t+1}\times TC^{t,t+1} \end{array}$$ (21)

The three input indicators and the expected output indicator for 2023–2030 can be estimated using the average annual growth rate from 2014 to 2022, while carbon emission data is derived through iterations of the allocation formulas under each principle. Since higher green productivity reflects efficient resource use, reduced environmental impact, and increased economic benefits—key goals of high-quality development—our focus will be on comparing the absolute values and relative trends of the GML index under both principles. This comparison will help identify the quota scheme most conducive to achieving high-quality economic development in China.

#### 4.3.2. Calculation results.

The results of the annual calculations for GML, EC, and TC can be found in Table 9. Comparing GML values across different years shows that green productivity growth based on the two principles during the “13th Five-Year Plan” period was significantly higher than during the “12th Five-Year Plan” period. If carbon quotas between provinces are allocated using AMLC principles, it is estimated that the average annual growth rate of green productivity during the “13th Five-Year Plan” period will reach 1.25%. This change indicates that AMLC quota allocation schemes will play a more critical role in guiding resources towards efficient and low-carbon production sectors in future high-quality development processes, promoting comprehensive improvement

**Table 9:** GML calculation and decomposition results based on the equity principle and AMLC principle (2023–2030).

Indicator name	Principle name	2023~ 2024	2024~2025	Average growth rate	2025~2026	2026~2027	2027~ 2028	2028~ 2029	2029~2030	Average growth rate	2023–2030 average value
GML	Equity	1.01	1.01	0.22%	1.01	1.02	1.02	1.03	1.04	0.68%	1.02
	AMLC	1.01	1.02	0.71%	1.03	1.03	1.03	1.05	1.08	1.25%	1.03
EC	Equity	1.00	1.00	0.25%	1.01	1.01	1.01	1.02	1.03	0.57%	1.01
	AMLC	1.01	1.01	−0.43%	1.01	1.02	1.01	1.01	1.01	−0.10%	1.01
TC	Equity	1.01	1.01	−0.03%	1.01	1.01	1.01	1.01	1.01	0.11%	1.01
	AMLC	1.00	1.01	1.10%	1.03	1.03	1.03	1.05	1.08	1.46%	1.03

of green economy nationwide. Overall, both equity principle and AMLC principle have had GML values greater than 1 over the years. The latter’s average value (1.03) is 0.01 higher than that of former (1.02), indicating that implementing both distribution schemes can have positive effects on national green productivity growth; however, implementing AMLC principle has a more significant impact on enhancing green productivity, demonstrating its optimal performance for high-quality development.

Since GML can be further decomposed into efficiency change EC and technological change TC components, analysis of data from Table 9 reveals: under equity principle, average value of EC indicator is 0.003 higher than under AMLC principle; however, this slight advantage cannot outweigh AMLC principle’s superior performance in TC indicator. This suggests that in future quota allocations, AMLC principle has greater potential to drive regional technological progress leading to broader improvements in green productivity levels.

## 5. Conclusions and recommendations

### 5.1. Conclusions

Under the total carbon reduction targets, regions play distinct roles. From 2003 to 2013, carbon flow in China mainly moved from carbon-exporting regions, rich in fossil fuels and dependent on external markets, to carbon-importing regions, lacking local fossil energy and with a single energy structure. This trend largely continued from 2014 to 2022, though Zhejiang, Hunan, Tianjin, and Beijing shifted from carbon-importing to carbon-exporting, further transferring emissions from developed to strategically aligned regions, exacerbating regional imbalances.

Existing research on formulating carbon emission allocation schemes primarily focuses on maximizing efficiency, prioritizing fairness, or adopting simple comprehensive allocation strategies. However, these studies have not fully considered the differences among regions in terms of economy, industry, and energy structure, which not only impact regional carbon emission capacities but also determine the responsibility division and policy response capabilities of various provinces in emission reduction tasks. To address these shortcomings, this paper identifies six clusters among China’s 30 provinces and municipalities through clustering analysis. Among them, the regional classification identified six clusters. Cluster 1 (Beijing, Shanghai) has the most rational industrial and energy structures. Cluster 2 (e.g., Fujian, Hainan) has the lowest historical carbon responsibility. Cluster 3 (e.g., Gansu, Guangxi) has the lowest income and the least favorable industrial and energy structures. Cluster 4 (e.g., Anhui, Hebei) has abundant agricultural carbon sinks but highly irrational energy consumption. Cluster 5 (e.g., Heilongjiang, Inner Mongolia) holds the largest forest carbon sinks, while Cluster 6 (Guangdong, Jiangsu, Shandong) bears the highest carbon responsibility and population density.

Allocation results differ between equity and efficiency principles. The equity principle favors Cluster 5 regions with carbon sinks, leading to quota surpluses in 17 regions, but it fails to benefit all clusters equally, with Cluster 6 regions facing quota deficits. Under the efficiency principle, 15 regions achieve quota surpluses. The mechanism benefits high-efficiency regions like Jiangsu and Guangdong, widening allocation inequalities and conflicting with China’s sustainability goals.

The AMLC principle allows 17 provinces and municipalities to achieve a quota surplus, with surplus regions distributed across all clusters, showing a stronger balance than the equity principle. However, it still struggles to alleviate the pressure faced by high carbon-emitting regions such as Shanxi, Shandong, and Liaoning. The first-stage allocation considers regional endowments and economic conditions while compensating high carbon-emitting regions, thereby tilting quotas towards Cluster 5 and 6 regions. The second-stage allocation ensures that resources are prioritized for regions within each group with higher marginal abatement costs. Additionally, the GML index average value from 2023 to 2030 based on the AMLC principle (1.03) is higher than the equity principle’s result (1.02), indicating that this principle more significantly enhances national green productivity and demonstrates the best performance in achieving high-quality development.

In summary, compared to the single-stage allocation principle proposed in existing research, the two-stage allocation principle presented in this paper fully considers the resource endowments and economic conditions of various regions in the first stage of allocation, providing compensation for regions with high carbon emission responsibilities. In the second stage, it ensures that resources flow to regions with the highest potential for efficient emission reduction. The advantage of this approach lies in its ability to effectively balance the resource flow relationship between high-carbon-emission clusters and high-efficiency-reduction clusters, avoiding potential problems that may arise from the pure pursuit of efficiency or fairness. This approach demonstrates greater flexibility and adaptability in practical applications.

### 5.2. Recommendations

Based on the above conclusions, we propose the following recommendations:

Under the equity principle, provinces rich in agricultural and forestry resources (such as Sichuan and Heilongjiang, primarily in Cluster 5) receive the highest quotas due to their strong carbon sequestration capacity. However, the significant surplus of quotas in these regions leads to resource wastage, while provinces with heavy historical carbon emission responsibilities (such as Guangdong and Shandong, mainly in Cluster 6) face substantial quota deficits. To address this issue, it is recommended that provinces in Cluster 5 leverage their advantages by actively developing and introducing low-carbon technologies, promoting green industrial transformation, and enhancing the economic value and utilization efficiency of their carbon sequestration resources. Additionally, the government should establish a more flexible quota trading mechanism, clearly map out the carbon transfer pathways between regions, and strengthen the incentives and regulatory measures for inter-regional carbon trading. This will ensure that surplus quotas in regions with excess capacity can be transferred to high-emission provinces, thus avoiding resource waste.

Under the efficiency principle, the gap between high-efficiency provinces (such as Jiangsu and Guangdong) and low-efficiency provinces (such as Sichuan and Henan) has widened, with the key focus being on improving the development efficiency of low-efficiency regions to narrow the gap. It is recommended to first establish a special fiscal support fund, with a focus on supporting low-carbon technology research and development as well as green industrial projects, to provide adequate financial guarantees for low-efficiency regions. Secondly, a green industrial investment fund should be established to encourage the participation of social capital, forming a development model that advances together with multiple parties. Meanwhile, tax incentives such as VAT exemptions and reductions, as well as halved corporate income tax, should be provided to enterprises adopting high-efficiency energy-saving equipment or utilizing renewable energy, in order to reduce their operating costs and stimulate their investment vitality. In addition, efforts should be made to promote cross-regional technical cooperation and talent exchange platforms, introducing advanced technologies and management experiences from high-efficiency provinces, facilitating technology sharing and experience learning, thereby comprehensively enhancing the overall competitiveness and development level of low-efficiency regions.

Although the AMLC principle better balances regional development needs and compensates provinces with high carbon emission responsibilities, regions with energy-intensive and heavy industrial sectors, such as Shanxi and Shandong, still face significant quota shortages. Therefore, the government should prioritize promoting the low-carbon transformation of resource-based provinces by taking technology transfer as a key lever and cultivating new forms of productive forces. This can be achieved by advancing industry-university-research cooperation and jointly launching green technology research and development projects. Additionally, regional technology sharing centers or technological innovation alliances should be established to serve as intermediaries for technology transfer between cross-regional enterprises. An intellectual property protection mechanism should be set up to ensure that technological achievements during the technology transfer process are properly protected, thereby motivating enterprises to invest more resources in technological research and development to maintain their competitive advantage. Furthermore, an effective market promotion mechanism should be designed to maximize the market value of green technologies.

Finally, relevant departments can efficiently classify regional development categories and solve quota allocations under the AMLC principle through software programming. To avoid path dependency, the results of the two-stage allocation should be adaptable to changing policy scenarios by updating data, adjusting indicators, and modifying weights in a timely manner to align with national development strategies. This principle can also provide a useful reference for the allocation of carbon emission rights among different cities, districts, and counties within provinces.

## Supporting information

S1 Appendix Composition of carbon emission indicators. (DOCX)

S1 Appendix Estimated carbon emission transfer levels of provinces and regions (2014–2022). (DOCX)

S1 Appendix Estimated national carbon emission quotas (2023–2030). (DOCX)
