# Supplementary material for: Optimizing China’s carbon quota allocation for peak emissions: A novel AMLC framework tailored to regional dynamics
Source: PLoS One. 2025 Apr 23;20(4):e0321644. doi: 10.1371/journal.pone.0321644 (PMC12017533; doi:10.1371/journal.pone.0321644)
Supplement: S1 Appendix — (DOCX) [file pone.0321644.s001.docx]

**Appendix 1. Composition of Carbon Emission Indicators.**

**Table 10. Carbon emission indicators.**

| Category | Raw coal | Coke | Crude oil | Gasoline | Kerosene | Diesel | Fuel oil | Natural gas |
| --- | --- | --- | --- | --- | --- | --- | --- | --- |
| CO_2_ emissions coefficients（tCO_2_/t） | 1.97 | 2.86 | 3.02 | 2.93 | 3.03 | 3.10 | 3.17 | 2.16^a^ |
| Standard coal conversion coefficients (kgce/kg) | 0.71 | 0.97 | 1.43 | 1.47 | 1.47 | 1.46 | 1.43 | 1.33^b^ |

Note: a is tCO_2_/1000Nm³, b is kgce/Nm³
