# Supplementary material for: Optimizing China’s carbon quota allocation for peak emissions: A novel AMLC framework tailored to regional dynamics
Source: PLoS One. 2025 Apr 23;20(4):e0321644. doi: 10.1371/journal.pone.0321644 (PMC12017533; doi:10.1371/journal.pone.0321644)
Supplement: S1 Appendix — (DOCX) [file pone.0321644.s002.docx]

**Appendix 2. Estimated Carbon Emission Transfer Levels of Provinces and Regions (2014–2022).**

**Table 11. Annual Average of PSO-LSSVM Model Feature Data from 2003 to 2013.**

| Region | Annual average of transferred carbon emissions | Annual average of economic development level $(\bar{x}_{1})$ | Annual average of population size $(\bar{x}_{2})$ | Annual average of industrial structure $(\bar{x}_{3})$ | Annual average of energy consumption structure$(\bar{x}_{4})$ | Annual average of technological innovation $(\bar{x}_{5})$ | Annual average of urbanization level $(\bar{x}_{6})$ | Annual average of openness level $(\bar{x}_{7})$ |
| --- | --- | --- | --- | --- | --- | --- | --- | --- |
| Liaoning | 1883.46 | 10549.56 | 4306.09 | 0.19 | 0.49 | 0.015 | 0.61 | 0.34 |
| Heilongjiang | 1707.69 | 6950.00 | 3795.91 | 0.31 | 0.34 | 0.010 | 0.55 | 0.16 |
| Shanxi | 1700.77 | 4854.49 | 3439.00 | 0.35 | 0.72 | 0.009 | 0.46 | 0.10 |
| Guangxi | 1603.20 | 5061.47 | 4750.45 | 0.32 | 0.53 | 0.005 | 0.38 | 0.12 |
| Shaanxi | 1255.15 | 5139.71 | 3726.00 | 0.43 | 0.49 | 0.022 | 0.42 | 0.08 |
| Henan | 1190.06 | 13540.44 | 9491.18 | 0.29 | 0.60 | 0.008 | 0.35 | 0.07 |
| Fujian | 1187.73 | 9824.38 | 3663.00 | 0.13 | 0.43 | 0.010 | 0.54 | 0.57 |
| Xinjiang | 1185.10 | 3347.24 | 2117.27 | 0.37 | 0.39 | 0.004 | 0.40 | 0.24 |
| Shandong | 1080.68 | 21864.42 | 9438.45 | 0.24 | 0.50 | 0.014 | 0.48 | 0.33 |
| Inner Mongolia | 797.68 | 5712.38 | 2435.36 | 0.44 | 0.59 | 0.004 | 0.52 | 0.07 |
| Hebei | 594.76 | 11335.16 | 7024.45 | 0.17 | 0.72 | 0.007 | 0.42 | 0.13 |
| Yunnan | 308.59 | 4609.12 | 4531.45 | 0.54 | 0.62 | 0.006 | 0.33 | 0.12 |
| Hubei | 203.78 | 9404.86 | 5725.73 | 0.34 | 0.58 | 0.014 | 0.47 | 0.10 |
| Anhui | 166.16 | 7629.49 | 6081.82 | 0.22 | 0.60 | 0.012 | 0.40 | 0.14 |
| Gansu | 130.47 | 2555.23 | 2547.55 | 0.52 | 0.48 | 0.010 | 0.34 | 0.11 |
| Ningxia | 95.26 | 796.16 | 620.64 | 0.38 | 0.55 | 0.007 | 0.46 | 0.10 |
| Jiangsu | 51.53 | 25240.45 | 7793.09 | 0.15 | 0.43 | 0.019 | 0.56 | 0.82 |
| Guizhou | -123.67 | 2758.41 | 3653.36 | 0.75 | 0.70 | 0.006 | 0.30 | 0.06 |
| Shanghai | -246.52 | 12112.27 | 2125.09 | 0.09 | 0.24 | 0.027 | 0.89 | 1.50 |
| Zhejiang | -284.06 | 18119.37 | 5270.36 | 0.09 | 0.29 | 0.016 | 0.59 | 0.62 |
| Jiangxi | -387.56 | 5529.75 | 4388.64 | 0.15 | 0.58 | 0.008 | 0.41 | 0.12 |
| Beijing | -531.35 | 9112.67 | 1780.36 | 0.04 | 0.27 | 0.055 | 0.85 | 1.42 |
| Hainan | -632.21 | 1313.97 | 858.64 | 0.18 | 0.16 | 0.003 | 0.48 | 0.27 |
| Guangdong | -911.87 | 30670.47 | 9991.00 | 0.18 | 0.25 | 0.015 | 0.63 | 1.32 |
| Sichuan | -989.43 | 10653.86 | 8127.27 | 0.23 | 0.48 | 0.014 | 0.38 | 0.12 |
| Tianjin | -1377.85 | 5098.28 | 1190.91 | 0.09 | 0.39 | 0.023 | 0.77 | 0.83 |
| Qinghai | -1797.42 | 724.72 | 554.55 | 0.37 | 0.35 | 0.006 | 0.43 | 0.06 |
| Hunan | -1833.67 | 9245.80 | 6501.00 | 0.21 | 0.59 | 0.010 | 0.41 | 0.07 |
| Jilin | -3167.51 | 4527.34 | 2717.64 | 0.30 | 0.53 | 0.010 | 0.53 | 0.14 |
| Chongqing | -8035.76 | 5366.84 | 2866.45 | 0.31 | 0.51 | 0.011 | 0.50 | 0.15 |
| National Average | -172.56 | 8788.28 | 4383.76 | 0.28 | 0.48 | 0.013 | 0.50 | 0.34 |

**Table 12. Annual Average of PSO-LSSVM Model Feature Data from 2014 to 2022.**

| Region | Annual average of transferred carbon emissions | Annual average of economic development level $(\bar{x}_{1})$ | Annual average of population size $(\bar{x}_{2})$ | Annual average of industrial structure $(\bar{x}_{3})$ | Annual average of energy consumption structure$(\bar{x}_{4})$ | Annual average of technological innovation $(\bar{x}_{5})$ | Annual average of urbanization level $(\bar{x}_{6})$ | Annual average of openness level $(\bar{x}_{7})$ |
| --- | --- | --- | --- | --- | --- | --- | --- | --- |
| Shandong | 5058.33 | 48366.15 | 10040.11 | 0.13 | 0.34 | 0.025 | 0.61 | 0.29 |
| Xinjiang | 4927.29 | 7766.82 | 2495.89 | 0.24 | 0.28 | 0.004 | 0.53 | 0.13 |
| Shanxi | 4291.10 | 9441.86 | 3502.33 | 0.24 | 0.58 | 0.010 | 0.60 | 0.08 |
| Anhui | 4267.42 | 19130.6 | 6067.89 | 0.14 | 0.43 | 0.024 | 0.55 | 0.14 |
| Zhejiang | 4047.02 | 39858.06 | 6261.11 | 0.04 | 0.15 | 0.031 | 0.70 | 0.52 |
| Fujian | 1895.41 | 24783.69 | 4087.44 | 0.08 | 0.28 | 0.023 | 0.67 | 0.37 |
| Shaanxi | 1810.25 | 12536.64 | 3910.11 | 0.39 | 0.43 | 0.026 | 0.59 | 0.13 |
| Jiangsu | 1628.90 | 59423.25 | 8423.56 | 0.06 | 0.32 | 0.031 | 0.71 | 0.46 |
| Hunan | 1591.26 | 23131.26 | 6625.56 | 0.19 | 0.45 | 0.019 | 0.56 | 0.09 |
| Tianjin | 1146.12 | 11112.36 | 1401.33 | 0.05 | 0.26 | 0.034 | 0.84 | 0.48 |
| Liaoning | 948.12 | 19523.39 | 4287.11 | 0.15 | 0.37 | 0.018 | 0.70 | 0.27 |
| Heilongjiang | 736.27 | 13402.73 | 3330.67 | 0.08 | 0.38 | 0.010 | 0.63 | 0.12 |
| Beijing | 533.69 | 18822.49 | 2188 | 0.02 | 0.04 | 0.056 | 0.87 | 0.84 |
| Inner Mongolia | 483.30 | 13158.56 | 2422.11 | 0.32 | 0.51 | 0.007 | 0.65 | 0.06 |
| Ningxia | 276.35 | 1820.18 | 707 | 0.29 | 0.53 | 0.014 | 0.62 | 0.07 |
| Qinghai | -184.09 | 1695.75 | 586.67 | 0.20 | 0.26 | 0.006 | 0.57 | 0.02 |
| Shanghai | -531.80 | 24934.06 | 2474 | 0.02 | 0.16 | 0.041 | 0.89 | 1.01 |
| Hainan | -560.03 | 3037.39 | 982.89 | 0.08 | 0.09 | 0.006 | 0.58 | 0.21 |
| Yunnan | -799.86 | 11552.7 | 4689.78 | 0.31 | 0.49 | 0.008 | 0.47 | 0.11 |
| Henan | -866.62 | 31644.08 | 9823.78 | 0.16 | 0.37 | 0.015 | 0.52 | 0.12 |
| Guangxi | -1052.18 | 11440.17 | 4930.78 | 0.22 | 0.43 | 0.006 | 0.52 | 0.21 |
| Gansu | -1182.45 | 5851.07 | 2511.44 | 0.35 | 0.35 | 0.012 | 0.49 | 0.06 |
| Hebei | -1630.54 | 23133.43 | 7406.33 | 0.11 | 0.59 | 0.014 | 0.57 | 0.12 |
| Guizhou | -2809.99 | 7562.69 | 3798 | 0.30 | 0.50 | 0.010 | 0.49 | 0.04 |
| Guangdong | -3258.63 | 67923.48 | 12224.22 | 0.09 | 0.19 | 0.048 | 0.72 | 0.75 |
| Sichuan | -3359.41 | 26470.75 | 8296 | 0.16 | 0.31 | 0.021 | 0.53 | 0.14 |
| Hubei | -3401.60 | 23109.63 | 5857.56 | 0.18 | 0.42 | 0.024 | 0.61 | 0.10 |
| Chongqing | -3711.14 | 14999.92 | 3150.22 | 0.15 | 0.32 | 0.022 | 0.66 | 0.28 |
| Jiangxi | -4972.97 | 14048.11 | 4507.22 | 0.10 | 0.45 | 0.019 | 0.57 | 0.16 |
| Jilin | -5336.34 | 8991.71 | 2489.11 | 0.22 | 0.38 | 0.010 | 0.61 | 0.10 |
| National Average | -0.56 | 19955.77 | 4649.27 | 0.170 | 0.36 | 0.020 | 0.62 | 0.25 |
