# Supplementary material for: Optimizing China’s carbon quota allocation for peak emissions: A novel AMLC framework tailored to regional dynamics
Source: PLoS One. 2025 Apr 23;20(4):e0321644. doi: 10.1371/journal.pone.0321644 (PMC12017533; doi:10.1371/journal.pone.0321644)
Supplement: S1 Appendix — (DOCX) [file pone.0321644.s003.docx]

**Appendix 3.** **Estimated National Carbon Emission Quotas (2023–2030).**

**Table 13.** **Estimated total national carbon emission quota from 2023 to 2030.**

| Period | Year | Total Carbon Emission Quota (Hundred Million Tons) |
| --- | --- | --- |
| 14th Five-Year Plan | 2023 | 132.14 |
|  | 2024 | 133.51 |
|  | 2025 | 134.90 |
| 15th Five-Year Plan | 2026 | 134.31 |
|  | 2027 | 133.72 |
|  | 2028 | 133.13 |
|  | 2029 | 132.54 |
|  | 2030 | 131.96 |
